# Elimination of Methicillin-Resistant *Staphylococcus aureus* from Mammary Glands of Dairy Cows by an Additional Antibiotic Treatment Prior to Dry Cow Treatment

**DOI:** 10.3390/microorganisms12122651

**Published:** 2024-12-20

**Authors:** Bernd-Alois Tenhagen, Mirka Elisabeth Wörmann, Anja Gretzschel, Mirjam Grobbel, Sven Maurischat, Tobias Lienen

**Affiliations:** 1 Department Biological Safety, German Federal Institute for Risk Assessment, 10589 Berlin, Germany; mirka.woermann@bfr.bund.de (M.E.W.); mirjam.grobbel@bfr.bund.de (M.G.); sven.maurischat@bfr.bund.de (S.M.); 2 Saxon State Laboratory of Health and Veterinary Affairs, 01099 Dresden, Germany; anja.gretzschel@lua.sms.sachsen.de

**Keywords:** MRSA, dry cow treatment, pirlimycin, skin colonization, dairy sector

## Abstract

Methicillin-resistant *Staphylococcus aureus* (MRSA) have been isolated from quarter milk samples of dairy cows, raising concerns over transmission to consumers of raw milk. This study investigates whether pre-treatment before dry-off can increase the success rate of dry cow treatment against MRSA. MRSA positive cows were assigned to two treatment groups. Both groups received dry cow treatment with a licensed product. The test group was additionally treated intramammarily with pirlimycin over seven days prior to the dry-off treatment. The use of pirlimycin increased the elimination of MRSA from previously MRSA positive udder quarters significantly (96.0 vs. 53.3%). However, MRSA were still present in noses and udder clefts of cows in MRSA negative quarter milk samples. New infections were observed in some quarters in both groups. Quarters that remained positive carried the same strain as prior to treatment. All MRSA isolates were associated with clonal complex CC398. Resistance to pirlimycin associated with the genes *erm*(C) or *lnu*(B) was observed in one isolate each from new infections after calving. Pretreatment supported the elimination of MRSA from the udder but did not eliminate MRSA from other body sites. Using the treatment will not eliminate the bacteria from the herd.

## 1. Introduction

Methicillin-resistant *Staphylococcus aureus* (MRSA) have been observed in livestock in the last 20 years. While in most food-producing animal species MRSA predominantly colonize skin and mucus membranes, in dairy cows MRSA have also been shown to be associated with clinical and subclinical mastitis [1,2,3,4]. Moreover, bulk tank milk of affected herds has been shown to contain MRSA, indicating that consumers of raw milk might be exposed to bovine MRSA [5,6,7]. Transmission of MRSA to humans via food in general is considered a minor issue, as the concentration of bacteria is typically low in meat. No data on MRSA concentration in milk have been published so far. Our own preliminary investigations resulted in most probable numbers of less than 10^3^ MPN/mL in bulk tank milk. However, the levels of MRSA in individual cows and quarters may exceed 10^3^ MPN/mL (unpublished data). This milk is unlikely to be consumed by humans undiluted but, when calves are fed on contaminated colostrum or milk, this might support colonization of calves, thus contributing to the spread of MRSA within a dairy farm. In line with this, milk fed calves have been shown to frequently harbor MRSA in positive dairy herds [5,8]. Moreover, clonal MRSA strains have been found in quarter milk samples, noses of calves and automatic calf feeders, indicating a transmission between cows and from cow to calf via milk feeding. Similar strains were also observed in humans, indicating occupational exposure to the bacteria [9].

Control of intramammary infection (IMI) in dairy cows with *S. aureus* using the five-point plan has largely been successful [10,11]. Dry cow treatment with antimicrobials plays an essential role in this control, as cure rates for intramammary *S. aureus* infections tend to be higher during the dry cow period than during lactation [12]. Moreover, treatment at or shortly before dry-off minimizes losses associated with milk withdrawal periods [13].

However, most dry cow products are based on beta-lactam antimicrobials and thus are not likely to be effective in the control of IMI with MRSA. Resistance of MRSA to other substances is also frequent, which further aggravates the situation. In this study, besides a dry-off therapy, which contains penicillin and an aminoglycoside, i.e., a substance that might be effective against beta-lactam resistant *S. aureus* [14], the additional use of pirlimycin was evaluated. Pirlimycin is a lincosamide approved for use in the treatment of subclinical IMI with *S. aureus* [15]. Resistance of *S. aureus* from IMI of dairy cows to pirlimycin varies between strains and countries. However, as compared to beta-lactams, resistance is rare in MRSA and resistance in *S. aureus* from IMI of dairy cows in Germany has been decreasing in recent years [16,17].

Another concern when using antimicrobials to control resistant pathogens is that this might lead to the acquisition of further resistance traits by the bacteria. Therefore, such approaches need thorough investigation regarding their efficacy and potential side effects.

It was, therefore, the objective of this study to carry out a preliminary investigation on the potential efficacy of two dry cow treatments in the control of intramammary infections with MRSA.

## 2. Materials and Methods

### 2.1. Dairy Herd and Dry-off Treatment

In a German dairy herd housing 800 lactating dairy cows with a known occurrence of MRSA [5], quarter milk samples were collected from all udder quarters about two weeks prior to dry-off. Between August 2020 and November 2021, cows with positive quarters were assigned to either treatment [n = 30] or control [n = 27] group by the practicing veterinarian. Ten treated and four control cows dropped out of the analysis because of missing data (see results chapter) leaving 20 and 23 for the analysis. Allocation was based on odd and even ear-tag numbers of the cows. Blinding was not considered feasible for farm staff due to the necessity of repeated intramammary treatments. However, laboratory staff analyzing the milk samples were not aware of allocation of the cows.

In the treated group, cows were treated intramammarily in all four quarters for seven days with pirlimycin (50 mg) (Pirsue^®^, Zoetis, Parsippany, NJ, USA). The control group did not receive this pre-treatment. At dry-off, after the last milking, cows in both groups were treated with a dry cow formulation containing framycetinsulfat (100 mg), benethamin-penicillin (280 mg) and penethamatehydroiodid (100 mg) (Benestermycin^®^, Boehringer Animal Health, Ingelheim, Germany), which has been shown to be effective against *S. aureus* [18]. As the study was carried out in a commercial herd and the herd did have issues with *S. aureus* infections, no untreated control group was included in the study. This implies that spontaneous cure rates of MRSA infections over the dry period could not be considered in the analysis. No explicit sample size determination was carried out beforehand. No further cows were included when, in an interim analysis, a significant difference regarding the main criterion (elimination of MRSA from previously infected glands) was noted at *p* < 0.01. Cows enrolled at that point in time completed the course of the study protocol.

### 2.2. Sampling

After calving, quarter milk samples of the cows from both groups were collected and tested for MRSA. For cows tested positive for MRSA on the same quarter as before, treatment was considered to have failed. Cows that tested negative after calving were re-tested after one to two months of lactation to determine whether they would remain negative. These cows were also sampled by collecting nasal swabs and udder cleft swabs to determine whether they were still carrying MRSA in other body sites. Nasal swabs were collected by rotating cotton swabs in the anterior nares of cows using the same swab for both nares. Udder cleft swabs were inserted between the skin of the udder and the hindleg and rotated likewise. Moreover, swab samples were taken from several cows that tested positive for MRSA in quarter milk samples after calving.

### 2.3. Culture and Confirmation

Culture and confirmation of MRSA was carried out by a two-step enrichment approach. Each swab sample (COPAN Diagnostics Inc., Murrieta, CA, USA) and 1 mL of each quarter milk sample was incubated in 10 mL (swab samples) or 9 mL of Mueller Hinton broth (Thermo Fisher Scientific Oxoid Ltd., Basingstoke, UK) supplemented with 6.0% NaCl (Merck, Darmstadt, Germany) for 24 ± 2 h at 37 °C. Of this pre-enrichment broth, 1 mL was transferred into 9 mL of tryptic soy broth (Merck, Darmstadt, Germany) supplemented with 3.5 mg/L of cefoxitin (Sigma-Aldrich, St. Louis, MO, USA) and 50 mg/L of aztreonam (Sigma-Aldrich, St. Louis, MO, USA). After incubation for 24 ± 2 h at 37 °C, selective-enrichment broth was plated onto chromogenic mannitol salt agar plates (Thermo Fisher Scientific Oxoid Ltd., Basingstoke, UK) containing 4 mg/L of cefoxitin (Sigma-Aldrich, St. Louis, MO, USA) and incubated for 24 ± 2 h at 37 °C. Depending on the colony morphology, one to three colony/colonies per positive chromogenic agar plates was/were sub-cultured onto sheep blood agar (Oxoid GmbH, Wesel, Germany). Identification of MRSA was conducted by real-time multiplex PCR analyzing the *tuf*, *nuc* and *mec*A genes using the primer and probes in Table 1 and the SensiFAST Probe No-ROX Kit (Bioline, Memphis, TN, USA).

### 2.4. WGS and Bioinformatics Analyses

All obtained MRSA strains were inoculated in 5 mL brain–heart-infusion broth (Merck, Darmstadt, Germany) and incubated at 37 °C for 24 h. DNA of 1 mL culture was extracted using the Qiagen DNeasy Blood and Tissue Kit (Qiagen, Hilden, Germany) according to the manufacturer’s protocol modified by adding 10 µL lysostaphin (Sigma-Aldrich, St. Louis, MO, USA) to the lysis buffer. The DNA library was prepared using an Illumina Nextera DNA Prep kit (Illumina Inc., San Diego, CA, USA) and the 150 bp paired-end sequencing run was performed on an Illumina NextSeq 500 instrument. Raw Illumina reads were trimmed and de novo assembled with the in-house developed AQUAMIS pipeline [19]. Bacterial characterization was conducted with the in-house developed Bakcharak pipeline (https://gitlab.com/bfr_bioinformatics/bakcharak, accessed on 8 February 2023) using the NCBI AMRfinder database [20] for screening of antimicrobial resistance (AMR) genes. Moreover, software tools from the Center for Genomic Epidemiology (https://cge.food.dtu.dk/services/ accessed on 1 February 2023) were applied. SCCmecFinder 1.2 [21,22,23] was used for analyses of SCC*mec* types and *spa*Typer 1.0 [24] was used for determination of *spa* types. *Spa* and SCC*mec* types were confirmed by PCR analysis and Sanger sequencing, as described in Schnitt et al. [5]. SNP analysis of MRSA strains was conducted using CSI Phylogeny 1.4 [25].

### 2.5. Antimicrobial Susceptibility Testing

Antimicrobial susceptibility testing was performed on a subset of isolates by broth microdilution according to ISO 20776-1:2006 [26] resp. methodologically consistent CLSI M07 Ed 11 [27]. It was carried out using a standardized antibiotic panel (Sensititre EUST scheme, ThermoScientific, Waltham, MA, USA), as recommended by the European Food Safety Authority (EFSA) for resistance monitoring in MRSA from livestock and food [28]. For interpretation of the minimum inhibitory concentration (MIC) of the individual strains, the EUCAST ECOFFs for *S. aureus* were used: penicillin > 0.125 mg/L; cefoxitin > 4 mg/L; chloramphenicol > 16 mg/L; ciprofloxacin > 1 mg/L; clindamycin > 0.25 mg/L; erythromycin > 1 mg/L; fusidic acid > 0.5 mg/L; gentamicin > 2 mg/L; kanamycin > 8 mg/L; linezolid > 4 mg/L; mupirocin > 1 mg/L; rifampin > 0.016 mg/L; sulfamethoxazole > 128 mg/L; streptomycin > 16 mg/L; quinupristin–dalfopristin > 1 mg/L; tetracycline > 1 mg/L; tiamulin > 2 mg/L; trimethoprim > 2 mg/L; vancomycin > 2 mg/L. The strain ATCC 29213 was used for quality control. In addition to the EUST scheme, pirlimycin resistance was tested using the Sensititre mastitis plate (Thermo Scientific, Waltham, MA, USA) interpreting pirlimycin resistance as a value of ≥4 mg/L.

### 2.6. Statistical Analyses

An infection was assumed if MRSA were detected in the quarter milk samples prior to dry-off. Successful treatment was defined by MRSA positive quarters prior to dry-off being negative after calving. The proportion of successfully treated quarters was compared between the two groups using a chi-square test. Positive quarters after sampling that had been negative prior to dry-off were considered as new infections.

## 3. Results

### 3.1. Efficiency of Pirlimycin Treatment

A total of 55 quarters from 43 cows with a history of MRSA detection in at least one udder quarter was included in the study. One cow was included twice in two consecutive lactations in the course of the study, once in the control and once in the treated group. Another 12 cows were included but dropped out of the analysis because the post-partum sample was missed (2 control, 5 treated cows), not taken because of clinical mastitis before the sample (1 cow each), or cows were not pregnant at dry-off (3 treated). One cow in each group was excluded for other reasons.

Of the included cows, 20 cows with a total of 25 MRSA-quarters were assigned to the treatment and 23 cows with 30 positive quarters to the control group. The positive quarters were equally distributed across the four quarters of the cows (15/14/13/13). In both groups, some cows had more than one positive quarter. This was observed in four cows from the control group that had four (1 cow), three (1 cow) and two (2 cows) positive quarters. In the treated group, five cows had two positive quarters each.

**Figure 1:**
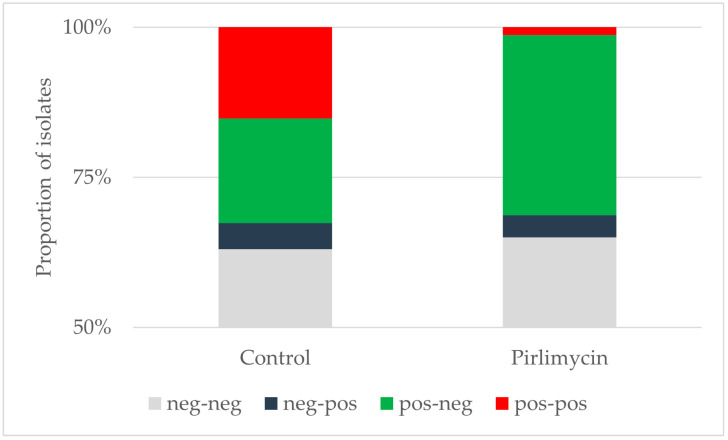
Status of udder quarters before dry-off and their respective diagnosis after parturition. More than half of the udder quarters were negative for MRSA prior to dry-off and after calving. Therefore, only the percentage range above 50% is displayed. neg-neg, negative prior to treatment and after calving; neg-pos, negative prior to treatment and positive after calving; pos-neg, positive prior to treatment and negative after calving; pos-pos, positive prior to treatment and after calving.

In total, more than half of the quarters across both groups were negative for MRSA prior to dry-off as well as after calving (Figure 1). Among the 30 MRSA positive quarters in the control group prior to dry-off, 16 (53.3%) were negative after calving. In the group pre-treated with pirlimycin, 24 of 25 previously MRSA positive quarters (96.0%) were negative after calving. The difference was significant (chi-square 12.52, *p* < 0.001). Among the cows with more than one positive quarter, six of eleven quarters in the control group (54.5%) and nine of ten quarters in the treated group (90.0%) were cured. In both groups, some quarters that had been negative prior to treatment, were positive after calving. This accounted for four quarters (6.4% of the negative quarters) in the control group and three quarters (5.5% of the negative quarters) in the treated group.

### 3.2. Typing Results

A total of 75 isolates were subjected to further typing, resistance testing and whole genome sequencing. Of these, the majority were assigned to *spa* type t034 (60/75) (Table 2). Six isolates were assigned to *spa* type t011, four to t588, three to t571, one to t19084 and one isolate could not be *spa* typed. In the treated group, 22/23 isolates collected prior to dry-off were assigned to t034, and one could not be typed. After calving, one of the four positive quarters harbored a t011, and the others harbored t034 isolates. In the control group, more diversity was seen, both before dry-off and after calving. All isolates carried the SCC*mec* type V and the *mecA* gene.

### 3.3. MRSA Colonization of Other Body Sites

To evaluate their MRSA status in other body niches, in addition to quarter milk samples, nasal and udder cleft swab samples were taken from 15 cows that had positive udder quarters prior to dry-off but were tested negative for MRSA in quarter milk samples after calving. Eight of these cows had been treated with pirlimycin. From the 15 cows, five cows carried MRSA only in the nose, two cows were positive for MRSA only in the udder cleft and eight cows carried MRSA in both nose and udder cleft. Moreover, nasal and udder cleft swabs of seven cows that tested positive for MRSA in the udder after calving were collected. Four of these cows carried MRSA only in the nose, two only in the udder cleft and one cow was colonized in both nose and udder cleft.

### 3.4. Antimicrobial Susceptibility Testing and Genotypic Characterization

Minimum inhibitory concentrations of the 75 isolates to 20 antimicrobials are displayed in Table 3. Two isolates were considered resistant to pirlimycin with an MIC above 4 mg/L. Both isolates were collected after calving, one in the treated and one in the control group. Both isolates were identified in previously uninfected quarters.

Several AMR genes were detected through WGS analysis in all strains. The AMR gene profile was similar in most strains and characterized by the genes *blaZ*, (penicillin resistance), *dfrG* (trimethoprim resistance), *mecA* (cefoxitin resistance), *tet*(K), *te*t(M) (tetracycline resistance) and *vga*(A) (tiamulin resistance). Other genes were only observed in individual isolates. The *erm*(C) gene was detected in one of the pirlimycin-resistant isolates, which illustrates an *erm*(C) resistance gene penetrance rate in the dairy herd of only 1% (1/75 isolates). This isolate was additionally resistant to clindamycin, erythromycin, quinupristin–dalfopristin, tiamulin, tetracycline and trimethoprim. The other pirlimycin-resistant strain harbored the *ant*(9)-Ia, *fexA*, *lnu*(B) and *lsa*(E) genes. The *lnu*(B) resistance gene penetrance rate, which confers lincosamide resistance, was also only 1%. The isolate was phenotypically resistant to clindamycin, chloramphenicol, quinupristin–dalfopristin, tiamulin, tetracycline, trimethoprim and pirlimycin. The *aadD1* gene was found in one strain, and this strain was resistant to kanamycin. 

### 3.5. Phylogenetic Analysis of MRSA Isolated from Quarter Milk Samples Before and After Dry-off

**Figure 2:**
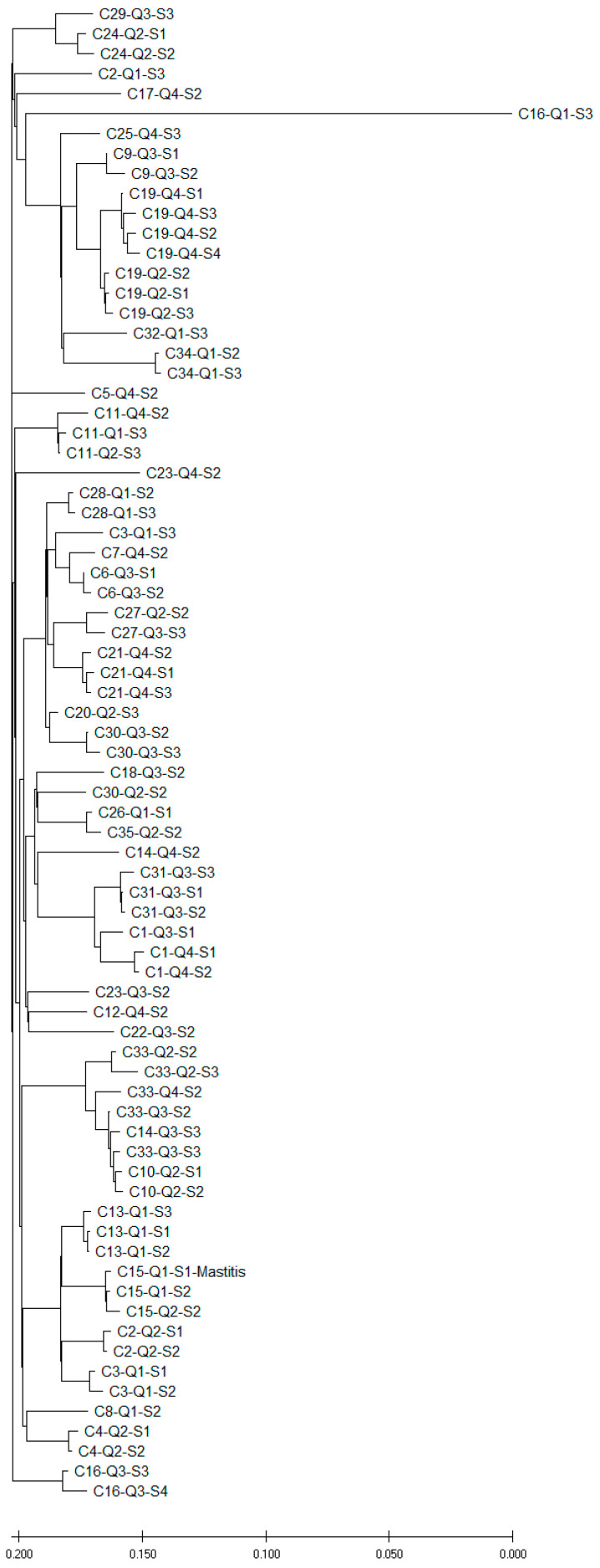
Genomic comparison (SNP analysis) of MRSA obtained from quarter milk samples of different cows (C), quarters (Q) and time points (S1, S2, S3, S4). S1 and S2 represent sampling before dry-off. Collection of S3 and S4 was carried out after calving. Cow C15 was affected by mastitis during the trial.

A comparison of the genomes of the MRSA strains by SNP analysis revealed a variety of different strains across the dairy herd (Figure 2). Isolates of MRSA from the same cow and quarter but at different time points S1, S2 (before dry-off) or S3 and S4 (after dry-off) clustered closely together in most cases. Whenever isolates were available from the same quarter before and after calving, isolates carried the same spa types and SCC*mec* types and the same set of resistance genes. In the phylogenetic tree, these isolates always clustered closely together. This persistent carriage of MRSA in the same udder quarter before dry-off and after calving was shown for the cows C13 (Q1), C19 (Q2 and Q4, respectively), C21 (Q4), C28 (Q1), C30 (Q3), C31 (Q3), C33 (Q2) and C34 (Q1). Most of these pairs were observed in the control group, as only in one quarter of the treated group did MRSA persist (C21, Q4). However, in this cow also, the same isolate was found after calving as before the treatment.

In some cows, different quarters of the same cow were affected by more distantly related MRSA strains. This was observed in cows C2 (Q1/Q2), C3 (Q1/Q4), C14 (Q3/Q4), C16 (Q1/Q3), C23 (Q3/Q4) or C30 (Q2/Q3). However, some cows also carried more closely related strains in different quarters [C1 (Q3/Q4), C11 (Q2/Q4), C13 (Q1/Q2) or C27 (Q2/Q3)]. Closely related MRSA strains were sometimes also found in quarter milk samples from different cows (C10/C14/C33 or C26/C35).

Overall, most of the positive quarters after calving had been positive before dry-off (15/22) and, in all quarters where isolates were compared, the infections were persistent (10/10). However, seven quarters were newly infected and, for those in the majority of cases (4/5), the isolates differed from those prior to calving. Therefore, they did not originate from the quarter that was positive before dry-off, but from another source.

Sometimes, new strains occurred in other quarters, while the originally positive quarters were cured. For example, in cows C2 and C3, one quarter was positive before dry-off and negative after calving, but the newly colonized quarter was positive after calving with a different strain. For two new cases, isolates could not be compared as the isolates prior to dry-off were not submitted for typing. However, the isolates of the new IMI in cow C3 showed some similarity to other isolates from other cows (C6 and C7, Figure 2). In cow C16, one quarter was positive prior to dry-off and two quarters were positive after calving. In this case, two different isolates were observed in the positive quarters post-partum, indicating that the newly infected quarter did not obtain the isolate from the treated quarter. In that case and in cow C2, they also differed from strains of other cows. To complete the mosaic, in cow C27, the positive quarter prior to dry-off was cured while a new quarter harbored the same strain after calving.

In cows C11 and C25, the colonization of the udder quarters by the respective MRSA strains changed over time. Interestingly, one MRSA strain from cow C16 (Q1) differed widely from all other strains.

### 3.6. Phylogenetic Comparison of MRSA from Nasal and Udder Cleft Swabs with MRSA from Quarter Milk Samples

The genomic comparison of all MRSA strains, which were isolated from quarter milk samples and swab samples from udder clefts and noses, revealed distantly related MRSA strains. Even in cows that carried MRSA in the udder, nose and udder cleft, the MRSA strains differed widely from each other.

## 4. Discussion

The use of antimicrobial treatment to control colonization and infection in animals is controversial because of the risk of promoting the development and spread of antimicrobial resistance. On this particular dairy farm, pirlimycin had occasionally been used to control *S. aureus* mastitis and MRSA colonization and was considered a successful approach. Results were, however, never evaluated systematically and were not compared to a non-pirlimycin-treated control group. In this study, whether such an approach could be effective in dairy cows was investigated, along with whether the debate on this issue is reasonable. If the treatment turned out to be non-effective, debate about its overall use would be futile. Pirlimycin is not among the highest priority critically important antimicrobials according to the WHO, or category A and B substances as designated by the European Medicines Agency (EMA) [29,30] and therefore the public health risk resulting from this trial was considered limited.

In this study, we found a significant reduction in the prevalence of MRSA positive udder quarters after calving as compared to the pre-dry-off phase. This is in line with previous studies supporting the effect of antimicrobial dry cow treatment on *S. aureus* infection in dairy cows [11,31]. Prolonged treatment with pirlimycin further reduced the number of positive quarters after calving. Only 1 of the 25 quarters that were positive prior to the dry cow treatment with pirlimycin was positive after calving, while 14 of 30 were MRSA positive after dry cow treatment only. Elimination of MRSA from cows in the control group might be caused by the aminoglycoside component of the dry cow product used. A spontaneous cure rate, independent of treatment, could not be considered in the analysis, as no untreated control cows were included. However, as dry cow treatment of quarters infected with *S. aureus* is still also standard in selective dry cow therapy, we did not consider this as relevant. Resistance to aminoglycosides was also rare in the MRSA isolates, with only one isolate being resistant to kanamycin and none to streptomycin or gentamicin. The improved elimination using pre-treatment with pirlimycin is in line with previous studies showing the efficacy of prolonged treatment with pirlimycin in controlling non-clinical *S. aureus* infections in udder quarters of dairy cows [32,33].

In both groups, a number of quarters were positive after calving which had been negative prior to dry-off. Interestingly, in most cases the quarters were colonized by a strain that differed from the strain that had been eliminated from another quarter of the cows. This was obvious for four cows, where isolates from prior to treatment and after calving were available for typing. In one cow, the strain just changed the quarter, i.e., it was found in the left hindquarter before treatment and in the right hindquarter after calving. For the other two strains, the respective isolates from the other quarter of the same cow prior to treatment were not available for typing. The reason for the new colonization could be twofold. One potential reason is the failure to detect these MRSA prior to dry-off. However, in using a highly sensitive detection method in this study, this seems a fairly unlikely event. The more likely scenario is that the cows were infected at some time between the dry-off treatment and testing after calving, e.g., in the dry cow group, the calving pen or the fresh cow group. This cannot be excluded and similarities between the isolates from some of the new infections and isolates from other cows render a transmission from these other cows likely. However, this was not the case in cows C2 and C16, where the isolates differed from those of other cows. Re-testing the cows that were negative after calving confirmed that, with one exception, the quarters did not harbor MRSA any longer. The exception was a cow that was negative after calving but harbored MRSA in the same quarter about half a year later. This strain, however, differed strongly from the previous strain and, therefore, was probably a new infection.

For the majority of cows which had MRSA positive quarters before treatment, but tested negative after calving, udder cleft or nose samples collected at re-testing were positive for MRSA. This underlines that the treatment will not eliminate MRSA from the animals or the herd directly, but only from the mammary gland of treated cows. This is not surprising: Intramammary treatments are expected to only have a very minor effect on the microbiota outside the mammary gland, as most of the drug will remain within the mammary gland. Interestingly, the isolates obtained from other body sites differed from those found in the quarter milk samples of the cows, indicating that spread of the MRSA in the environment may follow a different pattern.

The aim of the pirlimycin treatment, on the one hand, was to eliminate MRSA from the infected mammary gland, avoiding the need to cull infected cows. As, in some herds, MRSA may be found in a major proportion of the dairy herd [1,5], culling these animals is associated with massive costs. The results of our study are promising from this perspective, as elimination of MRSA from positive quarters was successfully induced. However, the effect is limited to the mammary gland and therefore this cannot be considered an overall solution to the problem.

The second aspect was to prevent the transmission of MRSA from cow to calf through colostrum. Calves have recently been shown to be the main carrier of MRSA in dairy herds [5] and transmission to MRSA via colostrum seems to be one major route [9]. Curbing carriage of MRSA in the mammary gland of pre-fresh cows might therefore help preventing MRSA in their calves. The obvious alternative of heat-treating colostrum to prevent MRSA transmission is hampered by the susceptibility of immunoglobulins to heat treatment. Temperatures up to 60 °C have been shown not to interfere with immunoglobulin-quality in colostrum [34]. However, 60 °C failed to eliminate MRSA from raw milk completely, although the treatment reduced MRSA counts in raw milk and colostrum substantially [35]. So far, it is unknown whether the colonization of calves is due to the feeding of contaminated raw milk and/or colostrum or originates from mother to offspring colonization during calving, as the cows have also been shown surface contamination. Further detailed studies around parturition are required in this context. Therefore, the treatment tested here only makes sense if it is embedded in a strategic approach to control MRSA (or *S. aureus* per se) in the herd, including milking hygiene, culling of chronically infected cows, and housing and milking infected cows separately.

The variability of spa types and the phylogenetic analyses illustrate the diversity of MRSA strains on this single dairy farm. However, individual udder quarters tended to consistently harbor the same strain over time. In addition, the phylogenetic analysis showed that, within individual cows, different udder quarters, as well as other body niches, such as the udder cleft or the nose, may be colonized by several MRSA strains with different AMR profiles. Since several cows were colonized by the same MRSA strain, most probably transmission processes have taken place in the dairy herd, which is in line with the contagious nature of *S. aureus* as a mastitis pathogen [36].

The phenotypic AMR profile of the analyzed MRSA strains had a conserved genetic backbone of resistance to penicillin, cephalosporins, tetracycline, trimethoprim and tiamulin, supplemented with additional genes in individual isolates. Pirlimycin resistance occurred in two MRSA strains from quarter milk samples collected after calving in this field study. This was associated with an increased clindamycin resistance (MIC > 4 mg/L), which is another antibiotic belonging to the lincosamide class. The genetic background of the pirlimycin resistance and the concurrently observed other resistance pattern differed between the two isolates. In one isolate, pirlimycin resistance was associated with carriage of the *erm*(C) gene. The other isolate carried the *lnu*(B) along with other resistance genes *(ant(9)-Ia*, *fexA*, and *lsa*(E)). The overall lincosamide resistance gene penetration rate in this dairy herd was rather low. However, the genes *erm*(C) and *lnu*(B) are often located on plasmids [37] and may be transferred to other MRSA in the dairy herd. Other AMR genes might also be transferred between different staphylococci. Therefore, monitoring of MRSA and AMR capabilities on dairy farms is of vital importance, especially when trying to combat the infections using other antimicrobials.

### Limitations

The study design was not fully in line with the approved requirements for studies on *S. aureus* in dairy cows with respect to the sampling strategy and to randomization. It was carried out on a commercial farm as part of attempts to reduce MRSA in the dairy herd. The results, therefore, need to be interpreted cautiously and the study should be repeated in other herds with a more rigorous approach. Not all isolates were available for further typing and sequencing. However, wherever isolates were available, results were in line with the hypotheses (i.e., persistent infection in quarters, differences between skin, nasal and quarter milk sample isolates within the same cow).

The substantial difference we saw between the groups regarding persistence of infection over the dry period supports the assumption that the results would not differ substantially when using a more rigorous approach. Potential misclassification of quarters as infected or not infected when only testing them once or twice needs to be considered. However, the likelihood that a positive quarter detected after parturition is negative later on is small. Likewise, the likelihood that a positive quarter prior to dry-off was not truly positive was similar in both groups and is therefore probably only a minor source of bias. We attempted to minimize the risk of misclassification by increasing inoculum size and using selective isolation, which is prone to be far more sensitive than routine mastitis sampling, with an inoculation of 10 microliters per sample [8].

## 5. Conclusions

Elimination of pirlimycin-susceptible MRSA from mammary glands using extended treatment with pirlimycin prior to dry-off is feasible but has no obvious effect on MRSA colonization in other body sites. Careful consideration should therefore be given to the risk of aggravating the resistance problems by using ‘just’ another antimicrobial for the resistant strains. The approach should therefore only be used in the framework of a systematic control plan for *S. aureus* and should be accompanied by routine characterization of the MRSA and other pathogens in the herd.

## Figures and Tables

**Table 1:** Primer and probes for identification of MRSA isolates.

Target	Primer/Probe	Nucleotide Sequence
*tuf*	Tuf-P1	AAACAACTGTTACTGGTGTAGAAATG
	Tuf-P2	AGTACGGAAATAGAATTGTG
	Tuf-Probe	TCCGTAAATTATTAGACTACGCTGAAGC
*nuc*	Nuc-P1	GTTGCTTAGTGTTAACTTTAGTTGTA
	Nuc-P2	AATGTCGCAGGTTCTTTATGTAATTT
	Nuc-Probe	AAGTCTAAGTAGCTCAGCAAATGCA
*mecA*	MecA-P1	AAATATTATTAGCTGATTCAGGTTAC
	MecA-P2	CGTTAATATTGCCATTATTTCTAAT
	MecA-Probe	CAAGGTGAAATACTGATTAACCCAGTA

**Table 2:** *spa* typing results for 75 isolates from the study. All isolates carried an SCC*mec* type V.

	Control		Pirlimycin		Total	
*spa* Type	Prior	Post	Prior	Post	Prior	Post
t011	4	1	0	1	4	2
t034	21	14	22	3	43	17
t571	2	1	0	0	2	1
t588	2	2	0	0	2	2
t19084	1	0	0	0	1	0
Not available	0	0	1	0	1	0
Total	30	18	23	4	53	22

*spa* = staphylococcal protein A.

**Table 3:** Minimum inhibitory concentrations of antimicrobials for the 75 isolates from quarter milk samples in the study. Vertical bars display the epidemiological cut-off values as provided by EUCAST, used for the evaluation of isolates as susceptible and resistant.

MIC (mg/L)	0.016	0.03	0.06	0.125	0.25	0.5	1	2	4	8	16	32	64	128	%res
Antimicrobial															
Clindamycin				15	54	3	1			2					8
Pirlimycin						63	10			2					2.7
Ciprofloxacin					48	26	1								0
Erythromycin					52	21	1			1					1.3
Cefoxitin										5	48	22			100
Fusidic Acid						75									0
Gentamicin							75								0
Chloramphenicol									69	5			1		1.3
Kanamycin									74		1				1.3
Linezolid							72	3							0
Mupirocin						75									0
Penicillin					1				74						100
Rifampicin	75														0
Sulfamethoxazol													75		0
Streptomycin									58	17					0
Quinupristin/ Dalfopristin						28	45	2							2.7
Tetracycline												75			100
Tiamulin									5	70					100
Trimethoprim								1					74		98.7
Vancomycin							74	1							0

MIC = minimum inhibitory concentration, %res = % of isolates that were classified as resistant using the chosen cut-off indicated by the vertical bar in the table. Grey shading denotes the range of concentrations tested. Values outside the right margin of the grey shading denote the number of isolates that grew in the highest tested concentration.

## Data Availability

The assembled sequences of all MRSA strains in this study are deposited in NCBI under the BioProject ID PRJNA634452.
